# Tailoring of Circularly Polarized Beams Employing Bound States in the Continuum in a Designed Photonic Crystal Metasurface Nanostructure

**DOI:** 10.3390/nano14171405

**Published:** 2024-08-28

**Authors:** Chunhao Xu, Minghao Chao, Zhizhong Liu, Qingsong Liu, Wenjing Zhang, Lingyun Zhuang, Bo Cheng, Botao Jiang, Jietao Liu, Guofeng Song

**Affiliations:** 1 Institute of Semiconductors, Chinese Academy of Sciences, Beijing 100083, China; 2 College of Materials Science and Opto-Electronic Technology, University of Chinese Academy of Sciences, Beijing 100049, China; 3 Laboratory of Photonic Integrated Circuits, Xiong’an Institute of Innovation, Chinese Academy of Sciences, Xiong’an New Area, Baoding 071700, China; 4 Institute of Intelligent Photonics, Nankai University, Tianjin 300071, China

**Keywords:** BIC, circular polarization, photonic crystal

## Abstract

We propose a photonic crystal (PC) nanostructure that combines bound states In the continuum (BIC) with a high-quality factor up to 10^7^ for emitting circularly polarized beams. We break the in-plane inversion symmetry of the unit cell by tilting the triangular hole of the hexagonal lattice, resulting in the conversion of a symmetrically protected BIC to a quasi-BIC. High-quality circularly polarized light is obtained efficiently by adjusting the tilt angles of the hole and the thickness of the PC layer. By changing the hole’s geometry in the unit cell, the Q-factor of circularly polarized light is further improved. The quality factor can be adjusted from 6.0 × 10^3^ to 1.7 × 10^7^ by deliberately changing the shape of the holes. Notably, the proposed nanostructure exhibits a large bandgap, which significantly facilitates the generation of stable single-mode resonance. The proposed structure is anticipated to have practical applications in the field of laser technology, particularly in the advancement of low-threshold PC surface emitting lasers (PCSELs).

## 1. Introduction

Metasurfaces have garnered significant interest in the scientific community due to their compatibility with semiconductor processing fabrication techniques, coupled with their exceptional capabilities in manipulating electromagnetic fields [1,2,3]. Metasurfaces, typically composed of layered structures with periodic variations in refractive index, exhibit a multitude of functionalities, including phase-modulating [4,5], beam steering [6], focusing [7,8,9], polarization manipulation [10,11,12,13,14], and beam shaping [15,16,17]. Among the various metasurface designs, the photonic crystal (PC) slab is a special structure that has a rigorous periodic condition. Focusing on polarization control, multiple PC structures that can generate circularly polarized light are achieved by changing the parameters of the unit structure [11,12]. However, these structures are often characterized by a low Q-factor and exhibit limited tolerance for single-mode oscillation when applied to lasers.

Recently, BICs have been applied to PCs to design and achieve high Q resonances [18,19,20,21,22,23,24]. According to previous research [20], the PC structure with high Q resonance can reduce the threshold of laser emission. BICs are peculiar states that remain localized in the radiation continuum with lifetimes that diverge to infinity [25], which can be transformed into quasi-BICs by breaking the structural in-plane inversion symmetry and introducing perturbations. By utilizing this feature, some circularly polarized light emission structures with high Q-factors have been achieved [21,22,23,24]. Most structures that utilize BIC to achieve circularly polarized emission rely on the oblique incidence of light or the anisotropy of the structure [22]. Nevertheless, these structures continue to encounter challenges, including low integration, non-vertical radiation, and the complexities associated with etching processes.

In this work, we design a PC structure incorporating quasi-BICs converted from symmetry-protected BICs, to achieve circularly polarized beams. This structure is evolved from the *C*_3_ symmetric structure by introducing one type of perturbation, the tilting angle of holes *θ*, which supports a higher quality factor. Additionally, our proposed structure exhibits a significantly larger photonic bandgap in comparison to previously reported designs in the literature [11,12]. This enhanced photonic bandgap is advantageous as it facilitates the generation of more stable single-mode resonance. The proposed PC structure offers the ability to emit circularly polarized light while allowing for adjustment of the quality factor by modifying the shape of the holes. The proposed structure not only exhibits vertical emission beams and a straightforward manufacturing process but also distinguishes itself from other PC structures by supporting circularly polarized beams. These attributes render our design a promising candidate for the development of compact, highly integrated circularly polarized light sources in PCSELs.

## 2. Materials and Methods

**Figure 1:**
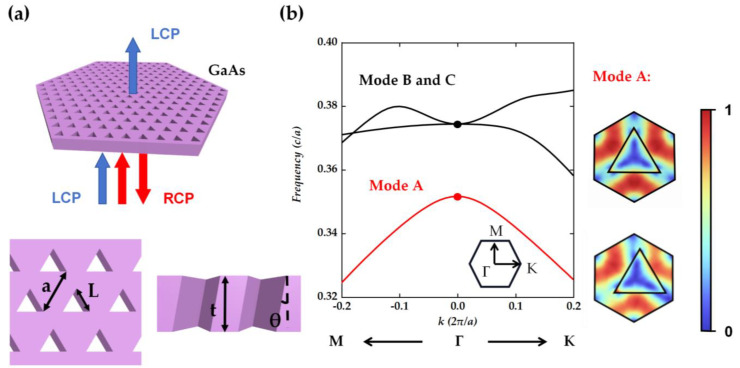
(**a**) Proposed metasurface with tilted holes for generation of circularly polarized beam. (**b**) The calculated photonic-band structure of the metasurface with triangular holes and electric field*/E/*distribution inside a unit cell at the top (upper panel) of the structure with a vertical/tilt hole. The thickness of the structure is 300 nm, and electric field distribution is obtained from eigenmode analysis.

The proposed structure of the PC is illustrated in Figure 1a, which consists of oblique triangular holes arranged in a hexagonal lattice. We consider the PC area to be infinite. As shown in Figure 1a, the left-handed circular polarization (LCP) will be transmitted, and the right-handed circular polarization (RCP) will be reflected. The structural parameters are set as *a* = 345 nm and *L* = 200 nm, where *a* and *L* refer to the lattice parameter and the side length of triangular holes, respectively. The proposed PC structure of GaAs (refractive index *n* = 3.55) layer (thickness *t*) is covered with PMMA (refractive index *n* = 1.48), to maintain the up-down mirror symmetry and avoid the influence of additional diffraction channels. The finite element method with COMSOL is used to calculate the electromagnetic field characteristics of the structure. Figure 1b shows the schematic of the photonic-band structure of the proposed PC layer. Focusing on the *Γ* point, the lowest frequency mode is labeled “Mode A”, and the corresponding wavelength is located at 980 nm. The subsequent calculation and research are based on the *Γ* point of “Mode A”. The first electric field in Figure 1b is the surface electric field for straight holes without tilt, while another pattern shows the surface electric field of the structure of holes with 10° tilted angles *θ*. Comparing the two electric fields, one can see that tilting the holes have a slight effect on the electric field of “Mode A”, and both cases have a significant enhancement of the electric field on the inner side/wall of the hole boundary. The main reason is the change in the magnitude of the electric field perpendicular to the dielectric constant boundary. There is a significant difference in refractive index between the two sides near the edge of the hole, where the vertical component of the electric field on the low refractive index medium side will significantly outweigh that on the high refractive index side [26]. The presence of holes can enhance the inside electric field, and the variation of the upper surface electric field significantly affects the far-field radiation. We can adjust the tilt angle and the shape of the holes, which allow us to control the phase difference between *E_x_* and *E_y_*. By doing this, we can further tailor the radiation properties, such as the amplitude characteristics of the emitted light.

**Figure 2:**
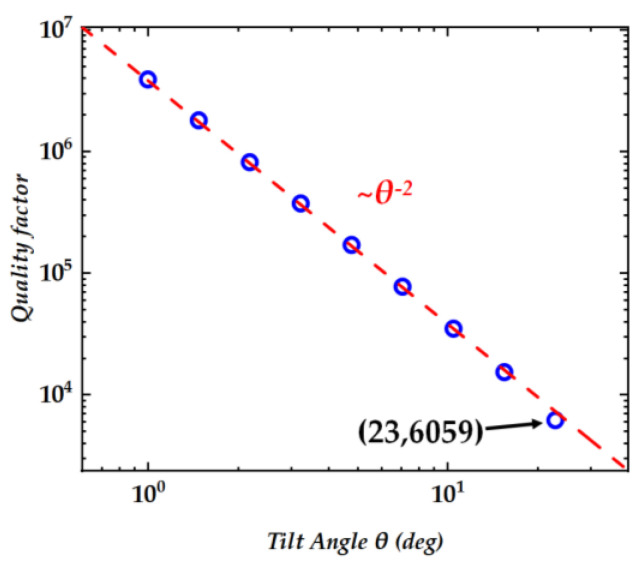
The relationship between the Q-factor and the tilt angle *θ*. The red dashed line is fitted to demonstrate the inverse quadratic dependence of *θ*. The thickness of the structure is set to 300 nm.

In addition, when the holes are vertical, the structure has *C*_3_ symmetry, which is due to the mutual cancellation of vertical radiation generated by the symmetrical positions. When the hole tilts, the *C*_3_ symmetry of the structure is disrupted, leading to the result that symmetrically protected BICs are converted into quasi-BICs. To quantify the relationship between radiation and the tilt angle, we calculated and plotted the relationship between Q-factor and tilt angles, as shown in Figure 2. The Q-factor represents the ability of light confinement, and in intrinsic mode analysis, the quality factor is equal to the ratio of the real and imaginary parts of the mode frequency. The tilt angle *θ* can be treated as the asymmetric factor, and the Q-factor is proportional to *θ*^−2^, which confirms that our structure can support high Q resonances arising from the distortion of symmetry-protected BICs (quasi-BICs). Employing an optical cavity with a high Q resonance significantly diminishes the laser emission threshold [20]. This attribute is advantageous as it facilitates a substantial reduction in the power consumption of laser systems, thereby enhancing their overall efficiency.

## 3. Results and Discussion

**Figure 3:**
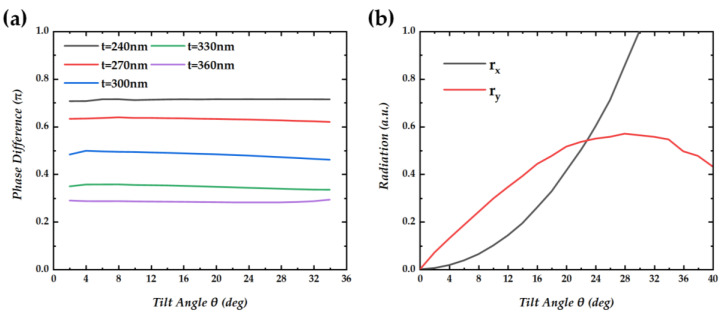

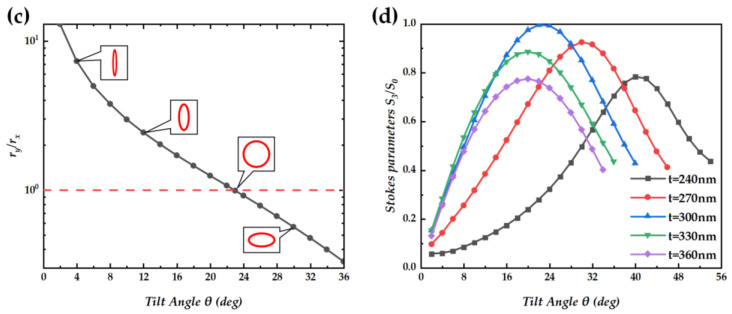
(**a**) Phase differences between *E_x_* and *E_y_* vs. tilt angle *θ*. (**b**) Normalized *r_x_* and *r_y_* vs. tilt angle *θ*. *r_x_* and *r_y_* refer to the radiation in *x* and *y* directions, respectively. (**c**) *r_x_*/*r_y_* vs. tilt angle *θ*. (**d**) Stokes parameter S_3_/S_0_, vs. tilt angle *θ* at the layer thicknesses of 240 nm, 270 nm, 300 nm, 330 nm, and 360 nm.

The results in Figure 3a show the variation of the radiation phase differences between *E_x_* and *E_y_* as a function of the tilt angle (2° to 34°) for varied PC-layer thicknesses *t*. One can see that the impact of the tilt angle on the radiation phase difference is slight, and the main factor affecting the radiation phase difference is the thickness *t* of the PC layer. When *t* = 300 nm, the phase difference between *E_x_* and *E_y_* reaches π/2, which is the condition for circularly polarized light. So, the thickness of the structure is set to 300 nm in the subsequent calculation and simulation. The result in Figure 3b shows *r_x_* and *r_y_* at different tilt angles in the case of *t* = 300 nm. In order to compare the radiation under different conditions more clearly, we normalized the radiation by taking the *x*-direction radiation *r_x_* as 1 at a tilt angle of 29°. *r_x_* and *r_y_* refer to the radiation in *x* and *y* directions, respectively, and the calculation method of *r_x_* and *r_y_* is shown in Appendix A. At *θ* = 0°, the structure exhibits *C*_3_ symmetry, and there is a symmetry-protected BIC resulting in zero radiation. As the tilt angle increases, the *r_x_* and *r_y_* increase at different speeds. As shown in Figure 3c, at a tilt angle of approximately 23°, the radiation amplitude in both directions is equal, achieving the condition of circular polarization. The result in Figure 3d shows the Stokes parameters of radiation light for different thicknesses and tilt angles. The radiation light achieves the maximum value of the Stokes parameter exclusively when the thickness is precisely set to 300 nanometers and the tilt angle is precisely 23 degrees. This specific combination of parameters is critical for optimizing the polarization characteristics of the emitted light. At this point, S_3_/S_0_ = 0.9978, and the Q-factor Q = 6.0 × 10^3^. The Q-factor here is low since the tilt angle required to achieve circularly polarized light is too large, which leads to excessive coupling with the radiation channel.

**Figure 4:**
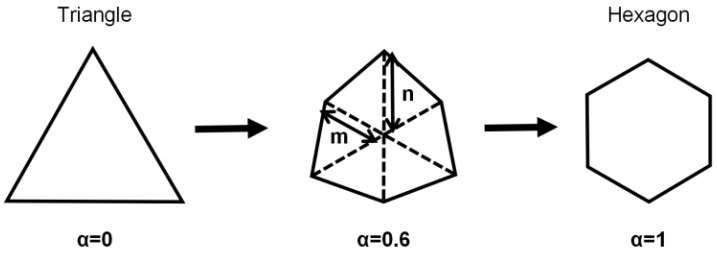
Definition of the parameter *α* and the designed shape of the holes.

To enhance the Q-factor of the proposed structure, we have engineered a novel configuration of holes. This design innovation effectively reduces the tilt angle necessary for the structure to emit circularly polarized light, thereby optimizing the performance of our system. Figure 4 shows the designed shape of the holes. We have maintained a constant PC slab thickness of 300 nanometers, a critical parameter that ensures a phase difference of π/2 between orthogonal polarizations. Furthermore, we have preserved the *C*_3_ symmetry within the structure, which is essential for the emergence of symmetry-protected BIC. We systematically transitioned the triangular holes to hexagonal configurations while meticulously maintaining the duty ratio constant. This approach is crucial to mitigating any unintended perturbations to the resonance frequency, thereby ensuring the stability of the optical properties throughout the morphological transformation. The shape parameter *α* = 2*m*/*n* − 1 represents the degree of change in shape, where *m* and *n* are the distances from the center to two vertices. At *α* = 0, the holes are triangular; and for *α* = 1, the holes are hexagonal.

**Figure 5:**
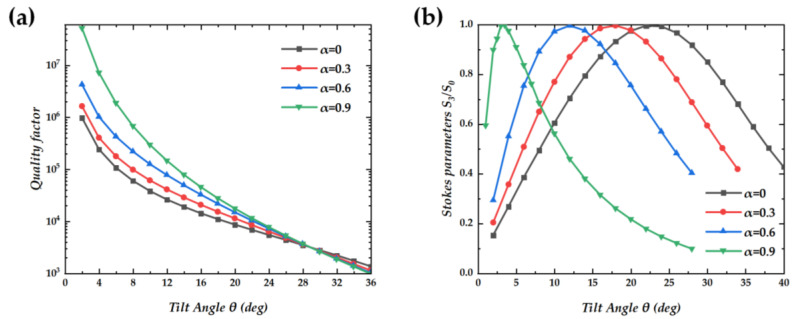
(**a**) The relationship between the Q-factor and the tilt angle *θ* at *α* = 0, 0.3, 0.6, and 0.9. (**b**) Stokes parameter S_3_/S_0_, vs. tilt angle *θ* at shape parameter of 0, 0.3, 0.6, and 0.9.

We calculated the impact of changes in hole shape on the Q-factor. As shown in Figure 5a, we chose four types of shapes (*α* = 0, 0.3, 0.6, and 0.9) to analyze the changes in quality factors, and all the Q-factors are approximately proportional to *θ*^−2^. At low angles, a subtle modification in the shape of the holes results in a modest enhancement of the structure’s Q-factor. This improvement is attributed to the higher symmetry-induced radiation cancellation, which effectively reduces the energy leakage from the resonant modes. Additionally, this phenomenon can be interpreted as an increase in the Q-factor in the vicinity of the *Γ* point. This enhancement is induced by the approach of the half-topological charge towards the *Γ* point [19,21,23], which alters the local density of states and consequently the resonant properties of the system. In addition, we investigated the influence of changes in hole shape on radiation polarization. Figure 5b shows the Stokes parameters at *α* = 0, 0.3, 0.6, and 0.9. The alteration in the shape of the holes diminishes the tilt angle necessary for the generation of circularly polarized light. This reduction in the required tilt angle is a direct consequence of the optimized hole geometry, which facilitates the coupling of light into the desired polarization state with greater efficiency. At *α* = 1, the radiation emanating from the inclined hole exhibits linear polarization.

**Figure 6:**
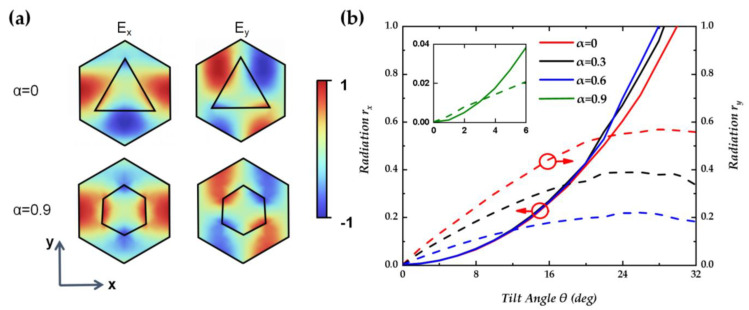

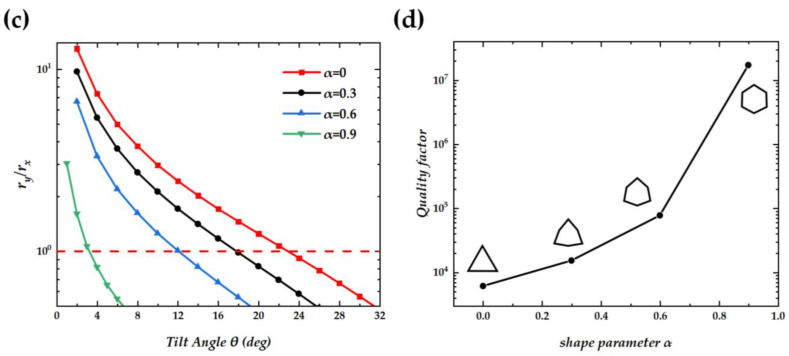
(**a**) Electric distribution field of *E_x_* and *E_y_* inside a unit cell at the top (upper panel) of the structure with different holes. (**b**) Normalized radiation of *r_x_* and *r_y_* vs. tilt angle *θ* at shape parameter of 0, 0.3, 0.6, and 0.9. (**c**) *r_x_*/*r_y_* vs. tilt angle *θ* at shape parameter of 0, 0.3, 0.6, and 0.9. (**d**) The quality factor of circular polarization vs. shape parameter *α*.

Figure 6a shows the electric field components of *E_x_* and *E_y_* in Mode A. Due to the significant difference in refractive index between the two sides near the edge of the holes, the vertical component of the electric field on the low refractive index side of the medium will be significantly greater than that on the high refractive index side, which is the reason that the holes can enhance the internal electric field. Consequently, holes at different positions exert distinct influences on the radiation characteristics, and we only need to pay attention to the holes and the variation in the electric field at symmetrical positions. It can be observed that there is a strong electric field of *E_x_* on the left and right sides of the hole. As the hole moves to the center of the strong electric field, the positive electric field *E_x_* is enhanced by the hole, breaking the previous situation of cancellation, and *r_x_* rapidly increases. Changing the hole shape enhances the symmetry of the *E_x_* electric field in the y-direction, which has little effect on the growth trend of *r_x_* as the holes move in the *x*-direction, as shown by the solid line in Figure 6b. In contrast to *E_x_*, the strong electric field center of *E_y_* is closer to the center of the cycle, which leads to an increase in *r_y_* and, correspondingly, reaching its maximum value earlier (*θ* = 26°). Changing the shape of the holes enhances the anti-symmetry of the *E_y_* electric field in the y-direction and greatly reduces the radiation *r_y_* intensity due to radiation cancellation, as shown by the dashed line in Figure 6c. Therefore, the tilt angle required for equal *r_x_* and *r_y_* is reduced due to the change in hole shape, and the low radiation intensity also means an improvement in quality factor. As shown in Figure 6c,d, at *α* = 0.9, the Q-factor value reaches 1.7 × 10^7^ at *θ* = 3°.

**Figure 7:**
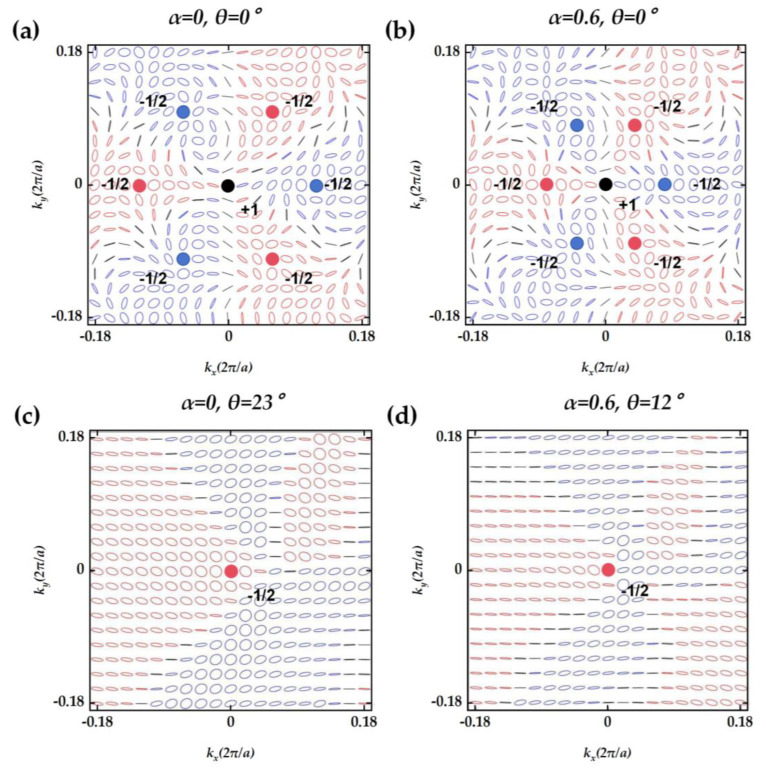
The momentum-space eigenpolarization map of Mode A for different *α* and *θ*. The red and blue dots represent right-handed and left-handed circularly polarized light with a topological charge of −1/2, respectively. The black dots represent the BIC with a topological charge of +1.

The momentum-space eigen polarization map of Mode A with shape parameter *α* = 0,0.6 is shown in Figure 7. At *α* = 0, a topological charge with υ = +1 is located at the *Γ*-point position in momentum space, with six symmetric circularly polarized states of υ = −1/2 around it, including three right-handed circular polarizations and three left-handed circular polarizations. The BIC with topological charge υ = +1 is the symmetric protected BIC, which originates from the *C*_3_ symmetry of the structure. As the shape of the holes progressively transforms into a hexagonal configuration, the circular polarization state in the polarization diagram gradually converges towards the *Γ*-point. According to a previous study [12], the inclination of pores will cause the overall polarization pattern to move in an inclined direction. Consequently, within the polarization diagram, the circular polarization state that is in proximity to the *Γ*-point corresponds to a reduced tilt angle and an elevated Q-factor. This correlation indicates that the closer the polarization state is to the *Γ*-point, the more efficiently the structure supports high Q resonances, which is associated with a lower tilt angle requirement for achieving circular polarization. When the holes transition to a fully hexagonal shape, the symmetry of the structure evolves from *C*_3_ to *C*_6_, indicating a higher order of rotational symmetry. Due to charge conservation, a topological charge of υ = +1 and six topological charges of υ = −1/2 are combined into a topological charge of υ = −2. During the process of topological charge merging, the Q-factor in the vicinity of the *Γ*-point experiences a significant enhancement, as depicted in Figure 5a. In contrast to other structures that rely on inclined holes to achieve circularly polarized light, our design leverages symmetry-protected BICs and the process of topological charge merging. This innovative approach not only facilitates the generation of circular polarization but also significantly enhances the Q-factor.

## 4. Conclusions

We proposed a novel PC structure capable of directly emitting circularly polarized beams. Our investigation has focused on optimizing the height and tilt angle of the triangular holes within the PC layer to achieve this polarization state. Furthermore, we have demonstrated the capability to attain high Q-factors by modifying the shape of these holes, thereby enhancing the resonance properties of the structure. Given the prerequisite of circularly polarized light emission, our study demonstrates that the quality factor (Q-factor) can be finely tuned within a range of 6.0 × 10^3^ to 1.7 × 10^7^ by strategically altering the shape of the holes. This tunability underscores the versatility of our photonic crystal structure in tailoring the resonance characteristics to meet specific optical requirements. Our research enables the selection of appropriate Q-factors tailored to specific applications, thereby achieving sufficient laser intensity and a reduced laser threshold. We have conducted a thorough analysis of the electric field distribution to elucidate the impact of hole shape on the optical properties of the structure. Recent advancements have indicated that the proposed PC structure is amenable to fabrication through the etching of tilted PC layers, as referenced in [12]. We anticipate that our contributions will significantly enhance the performance of circularly polarized lasers, thereby expanding their utility across a range of laser applications.

## Data Availability

Data underlying the results presented in this paper are not publicly available at this time but may be obtained from the authors upon reasonable request.

## Appendix A. Calculation of Stokes Parameters Using the Finite Element Method of COMSOL

By calculating the electric field size *E_x_*(*x*,*y*) and *E_y_*(*x*,*y*) of the output beam at z = 2500 nm, the complex radiation field *r_x_*, and *r_y_* above the PC can be determined by:

(A1) $$r_{x}=\frac{1}{S}\iint_{S}E_{x}(x,y)e^{-i\cdot (k_{x}\cdot x+k_{y}\cdot y)}dxdy$$

(A2) $$r_{y}=\frac{1}{S}\iint_{S}E_{y}(x,y)e^{-i\cdot (k_{x}\cdot x+k_{y}\cdot y)}dxdy$$

where *r_x_* and *r_y_* are the complex radiation fields above the PC surface, *k_x_* and *k_y_* are the wave vectors, *S* is the area of a lattice unit, and z = 0 is defined in the upper layer of the PC layer. The Stokes parameter can be expressed [27] as:

(A3) $$S_{0}={\left|r_{x}\right|}^{2}+{\left|r_{y}\right|}^{2}$$

(A4) $$S_{1}={\left|r_{x}\right|}^{2}-{\left|r_{y}\right|}^{2}$$

(A5) $$S_{2}=2\left|r_{x}\right|\left|r_{y}\right|\cos(\Delta \varphi )$$

(A6) $$S_{3}=2\left|r_{x}\right|\left|r_{y}\right|\sin(\Delta \varphi )$$

where Δ*φ* is the phase difference of radiation between *E_x_* and *E_y_*, which is calculated from the complex radiation field.
